# Sepsisassoziierte Todesfälle in Deutschland: Charakteristika und regionale Variation

**DOI:** 10.1007/s00103-021-03427-5

**Published:** 2021-11-08

**Authors:** Carolin Fleischmann-Struzek, Norman Rose, Konrad Reinhart

**Affiliations:** 1 Institut für Infektionsmedizin und Krankenhaushygiene, Universitätsklinikum Jena, Am Klinikum 1, 07740 Jena, Deutschland; 2 Center for Sepsis Control and Care, Universitätsklinikum Jena, Jena, Deutschland; 3 Klinik für Anästhesiologie m. S. operative Intensivmedizin, Charité Universitätsmedizin Berlin, Berlin, Deutschland

**Keywords:** Sepsis, Septischer Schock, Epidemiologie, Mortalität, Regionale Unterschiede, Sepsis, Septic shock, Epidemiology, Mortality, Regional disparities

## Abstract

**Hintergrund:**

Sepsis ist weltweit jährlich für geschätzt 11 Mio. Todesfälle verantwortlich. Die Epidemiologie sepsisassoziierter Todesfälle ist in Deutschland unzureichend verstanden, da Sepsis bisher nicht über die deutsche unikausale Todesursachenstatistik erfasst werden kann.

**Ziel der Arbeit:**

Epidemiologie und Charakteristika sepsisassoziierter Krankenhaustodesfälle sollen analysiert sowie regionale Unterschiede beschrieben werden.

**Material und Methoden:**

Retrospektive Beobachtungsstudie basierend auf der deutschlandweiten fallpauschalenbezogenen Krankenhausstatistik (DRG-Statistik) 2016. Sepsisassoziierte Krankenhaustodesfälle wurden über explizite und implizite Sepsis-ICD-10-GM(Internationale statistische Klassifikation der Krankheiten und verwandter Gesundheitsprobleme, 10. Revision, German Modification)-Codierungen identifiziert. Verstorbene wurden anhand der klinischen Merkmale und ihres Wohnortes entsprechend Amtlichem Gemeindeschlüssel (AGS-5-Steller) charakterisiert. Basierend auf der Bevölkerungsstatistik wurde der Anteil an den Gesamttodesfällen ermittelt.

**Ergebnisse:**

2016 gab es in Deutschland 58.689 mit explizit codierter Sepsis assoziierte Krankenhaustodesfälle (14,1 % aller Krankenhaustodesfälle). Die Mortalität betrug 73/100.000 Einwohner und variierte 1,8-fach zwischen den Bundesländern und 7,9-fach zwischen den Kreisen. 6,4 % der deutschlandweiten Todesfälle waren sepsisassoziierte Krankenhaustodesfälle. Dieser Anteil war am höchsten in der Altersgruppe der 40- bis 64-Jährigen (9,6 %) und höher bei Männern als bei Frauen (7,7 % vs. 5,2 %). Im Vergleich dazu betrug der Anteil von implizit codierten sepsisassoziierten Krankenhaustodesfällen 47,2 % an allen Krankenhaustodesfällen und 21,6 % an allen Todesfällen.

**Diskussion:**

Auch wenn die direkte Todesursache nicht sicher ableitbar ist, lässt sich aus dem hohen Anteil sepsisassoziierter Todesfälle an den Krankenhaustodesfällen der Bedarf weiterer Forschung und epidemiologischer Surveillance ableiten, zum Beispiel in Kohortenstudien oder auf Basis von multikausalen Todesursachenstatistiken.

**Zusatzmaterial online:**

Zusätzliche Informationen sind in der Online-Version dieses Artikels (10.1007/s00103-021-03427-5) enthalten.

## Abstract (en)

**Background:**

An estimated 11 million deaths are associated with sepsis worldwide. The epidemiology of sepsis-associated deaths in Germany is insufficiently understood, as sepsis deaths cannot be identified in the German mono-causal causes of death statistics.

**Aim:**

We aim to analyze the epidemiology and characteristics of sepsis-associated hospital deaths as well as to describe regional disparities.

**Materials and methods:**

Retrospective cohort study based on the 2016 German-wide diagnosis related groups (DRG) statistics. Sepsis-associated hospital deaths were identified by explicit and implicit sepsis ICD-10-GM codes. Deaths were described based on clinical characteristics and place of residence by official municipality key. The proportion of sepsis-associated hospital deaths among all deaths was calculated based on the national population statistics.

**Results:**

In 2016, there were 58,689 hospital deaths associated with explicitly coded sepsis (14.1% of all hospital deaths). Sepsis mortality was 73 per 100,000 inhabitants and varied 1.8-fold between federal states and 7.9-fold between districts. Of the national deaths, 6.4% were sepsis-associated hospital deaths. This proportion was highest in the 40–64 years age group (9.6%) and higher in males compared to females (7.7% vs. 5.2%). Compared to this, the proportion of deaths associated with implicitly coded sepsis was 47.2% among all hospital deaths and 21.6% among all national deaths.

**Discussion:**

Although the direct cause of death cannot be assessed based on our data, the high proportion of sepsis-associated deaths calls for further research and epidemiological surveillance, e.g., by cohort studies or based on multi-causal death statistics in Germany.

## Einleitung

Sepsis ist ein lebensbedrohliches Organversagen, das durch eine fehlgesteuerte Immunantwort des Körpers auf eine Infektion ausgelöst wird [1]. Sie kann zu Schock, Multiorganversagen und Tod führen, insbesondere wenn sie nicht früh erkannt und rechtzeitig behandelt wird [2]. Eine aktuelle Analyse der Global-Burden-of-Disease-Studie schätzt, dass weltweit 49 Mio. Menschen an Sepsis erkranken und 20 % der globalen Todesfälle mit Sepsis assoziiert sind [3]. Häufig tritt die Sepsis als Komplikationen nichtübertragbarer Erkrankungen und nach Unfällen auf [3]. Sie stellt auch die Gesundheitssysteme von Industriestaaten vor große Herausforderungen [4].

Todesfälle im Zusammenhang mit Sepsis und mögliche regionale Unterschiede sind in Deutschland bisher unzureichend erforscht. Sepsis wird in der Systematik der Todesursachen als „intermediär“ oder „unmittelbar zum Tod führende Erkrankung“ klassifiziert. Da nur das ursächliche Grundleiden in die deutsche Todesursachenstatistik einfließt („monokausale Aufbereitung“), kann eine direkte Abschätzung der sepsisassoziierten Todesfälle über diese Datenquelle nicht erfolgen. Eine erste Schätzung der sepsisassoziierten Todesfälle in Deutschland lieferte in 2020 die Global-Burden-of-Disease-Studie, in der die Daten multikausaler Todesursachenstatistiken aus 4 Ländern (USA, Brasilien, Mexiko, Taiwan) unter Adjustierung für den Zugang und die Qualität des Gesundheitswesens für andere Länder extrapoliert wurden. Die Studie schätzt für Deutschland 54.666 sepsisassoziierte Todesfälle im Jahr 2017 [3]. Die Genauigkeit dieser Schätzung ist allerdings noch unbekannt und durch die geringe Primärdatenbasis limitiert.

Da sich 87 % der Sepsistodesfälle im Krankenhaus ereignen [5], kann die Analyse von Krankenhaustodesfällen mit Sepsis zu einem besseren Verständnis sepsisassoziierter Todesfälle beitragen [6] und wird in vielen Ländern zur Erfassung der Sepsismortalität einer Population verwendet [7]. Im Jahr 2007 fanden sich, basierend auf einer vorausgegangenen Analyse der deutschlandweiten Krankenhausentlassdaten der fallpauschalenbezogenen Krankenhausstatistik (DRG-Statistik), 26.606 sepsisassoziierte Todesfälle [8]; bis 2015 hat sich diese Zahl auf 56.875 mehr als verdoppelt [9].

Ziel dieser Analyse ist es, basierend auf der Datengrundlage DRG-Statistik, sepsisassoziierte Krankenhaustodesfälle in Deutschland mittels unterschiedlicher Identifikationsmethoden zu analysieren und regionale Unterschiede zu beschreiben.

## Methodik

### Datengrundlage

Es handelt sich um eine retrospektive Beobachtungsstudie basierend auf Daten der deutschlandweiten DRG-Statistik des Statistischen Bundesamtes. Diese stellt eine Vollerhebung der Krankenhausbehandlungen aller deutschen Krankenhäuser dar, die nach dem DRG-Vergütungssystem abrechnen und dem §1 des Krankenhausentgeltgesetzes (KHEntgG) unterliegen. Nicht in der DRG-Statistik enthalten sind Daten von Krankenhäusern im Straf- oder Maßregelvollzug, Polizeikrankenhäusern sowie psychiatrischen und psychosomatischen Krankenhäusern. Auf die DRG-Statistik wurde mittels kontrollierter Datenfernverarbeitung zugegriffen. Weiterhin wurden Daten zu Sterbefällen aus der nationalen Bevölkerungsstatistik verwendet, die auf der Website des Statistischen Bundesamtes frei zugänglich sind (www.destatis.de).

### Studienpopulation

Ausgewertet wurden die Daten aller im Jahr 2016 in Deutschland vollstationär behandelten Patienten. Sepsisassoziierte Krankenhaustodesfälle wurden identifiziert als Krankenhaustodesfälle mit den folgenden expliziten ICD-10-GM(Internationale statistische Klassifikation der Krankheiten und verwandter Gesundheitsprobleme, 10. Revision, German Modification)-Sepsis-Entlassdiagnosen: R65.1! (Sepsis mit Organversagen) und R57.2 (septischer Schock). Diese ICD-10-GM-Codes wurden im Jahr 2016 nach den damals gültigen Sepsiskriterien der S3-Leitlinie Sepsis [10] in Deutschland definiert, die Sepsis ebenfalls wie die aktuell gültige Sepsisdefinition [1] als infektionsbedingtes Organversagen klassifizierte. Zum Vergleich wird in dieser Arbeit die implizite Identifikationsstrategie herangezogen (sogenannte Angus-Implementierung; [9, 11]), da sie im Gegensatz zur expliziten Identifikationsstrategie als weniger anfällig für Codiereinflüsse, z. B. externe Codieranreize, gilt [12]. Krankenhaustodesfälle mit implizit codierter Sepsis wurden als Todesfälle mit gleichzeitigem Vorliegen eines ICD-10-GM-Codes für Infektion und eines ICD-10-GM-Codes für Organversagen bei Krankenhausentlassung gewertet. Implizit erfasste Sepsisfälle schließen explizite Sepsisfälle mit ein.

### Messungen

Als Endpunkte wurden analysiert: Sepsiskrankenhaussterblichkeit und -mortalität pro 100.000 Einwohner sowie Anteil der sepsisassoziierten Krankenhaustodesfälle pro 100 Todesfälle in Gesamtdeutschland sowie auf Kreisebene (entsprechend Patientenwohnort entsprechend Amtlichem Gemeindeschlüssel (AGS-5-Steller)). Sepsisassoziierte Krankenhaustodesfälle wurden nach Alter, Geschlecht, Vorerkrankungen entsprechend ICD-10-GM-Diagnosen des Charlson-Komorbiditätsindex [13], Infektfokus entsprechend ICD-10-GM-Diagnosen (siehe Onlinematerial) und erfolgten Therapien (intensivmedizinische Komplexbehandlung, Beatmung, chirurgischer Eingriff nach OPS(Operationen- und Prozedurenschlüssel)-Codes, siehe Onlinematerial) charakterisiert.

### Statistische Analysen

Kategoriale Variablen werden hinsichtlich ihrer Häufigkeitsverteilung anhand von absoluten und relativen Häufigkeiten in Prozent in den jeweils betrachteten Populationen und Subpopulationen beschrieben. Die Berechnung der Inzidenz für Deutschland insgesamt sowie auf Ebene der Landkreise bzw. kreisfreien Städte (im Folgenden unter „Landkreise“ zusammengefasst) erfolgt anhand der absoluten Fallzahlen, die sich aus der DRG-Statistik ergeben im Verhältnis zur jeweiligen Einwohnerzahl gemäß der Angabe des Statistischen Bundesamtes. Die altersstandardisierten Zahlen inzidenter Sepsisfälle je Landkreis wurden anhand der direkten Altersstandardisierung berechnet, mit der Gesamtbevölkerung von Deutschland im Jahr 2016 als Referenzpopulation. Fälle, die in der DRG-Statistik nur eine gültige Bundesland-, aber keine Kreiskennzeichnung enthielten, mussten bei der Berechnung der Kennzahlen auf Kreisebene ausgeschlossen werden (ausgeschlossene Fälle mit expliziter Sepsis: *n* = 777, septischem Schock: *n* = 298, impliziter Sepsis: *n* = 5085).

Die Verteilung metrischer Variablen wird anhand des Medians, des Interquartilsbereiches sowie Maximums und Minimums charakterisiert. Die regionale Verteilung der Anteile sepsisassoziierter Krankenhaustodesfälle unter allen Todesfällen je Landkreis in Deutschland wurde kartografisch dargestellt. Die Analysen und die Erstellung der Grafiken erfolgten unter Verwendung von SAS/STAT® und R [14, 15].

## Ergebnisse

### Sepsisassoziierte Krankenhaustodesfälle (explizite Codierung)

**Figure 1:**
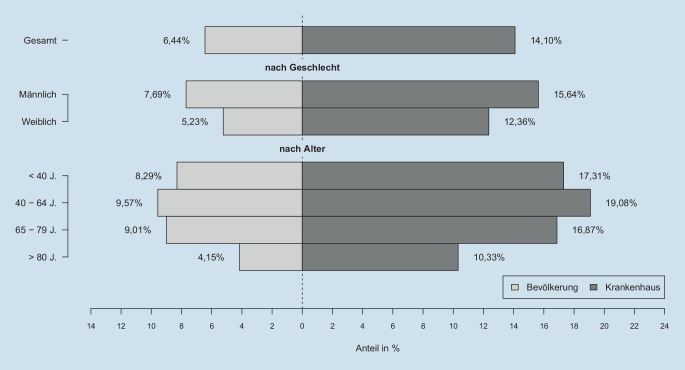

Unter 18,9 Mio. Krankenhausfällen in 1496 Krankenhäusern wurden 146.985 explizit codierte Sepsisfälle (0,8 %) identifiziert, von denen 44.657 (30,4 %) einen septischen Schock hatten. 39,9 % der Sepsispatienten (58.689) verstarben im Krankenhaus. Unter Patienten mit septischem Schock lag die Krankenhaussterblichkeit bei 56,2 %. Insgesamt wurde bei 14,1 % aller Krankenhaustodesfälle ein expliziter Sepsiscode bei Entlassung codiert (Abb. 1). In der Altersgruppe der 40- bis 64-Jährigen war dieser Anteil mit 19,1 % am höchsten, während er in der Altersgruppe > 80 Jahre bei 10,3 % lag.

**Figure 2:**
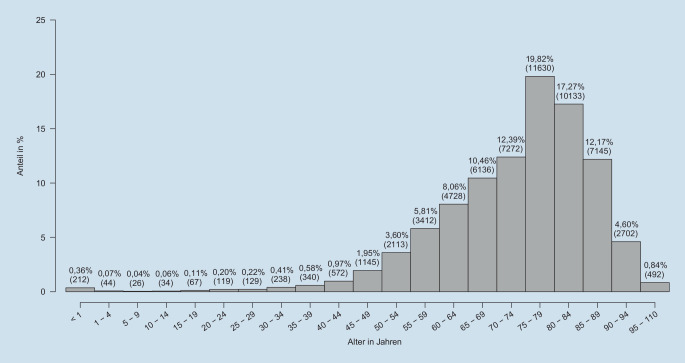

Die Verstorbenen waren im Median 76 („interquartile range“ [IQR] 16) Jahre alt (Altersverteilung Abb. 2), 41,2 % waren weiblich. Sie hatten im Median 2 (IQR 2) Komorbiditäten nach Charlson-Komorbiditätsindex, am häufigsten waren Herzinsuffizienz/Myokardinfarkt (45,4 %), renale Erkrankungen (33,5 %) und Diabetes mellitus (32,2 %). Von den sepsisassoziierten Krankenhaustodesfällen hatten 60,0 % während ihres Aufenthaltes eine intensivmedizinische Komplexbehandlung erhalten; 63,8 % waren mit einer medianen Beatmungsdauer von 119 (IQR 288) Stunden beatmet worden. 43,2 % der sepsisassoziierten Krankenhaustodesfälle waren nach einem chirurgischen Eingriff verstorben.

**Figure 3:**
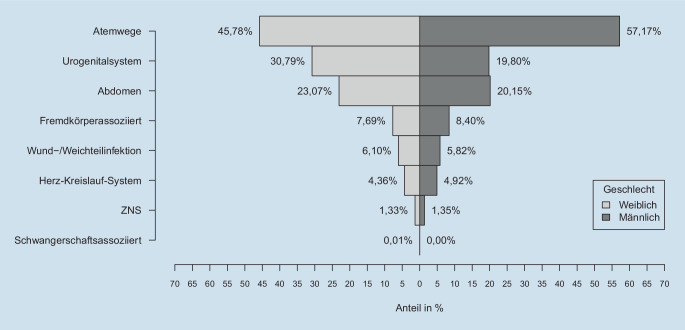

Häufigster Infektfokus waren die Atemwege (52,5 %) und das Urogenitalsystem (24,3 %), wobei ein Patient mehr als einen Fokus haben kann. Atemwegsinfekte als Sepsisfokus traten deutlich häufiger bei Männern als bei Frauen auf, während Infekte des Urogenitalsystems bei Frauen häufiger ein Sepsisfokus waren als bei Männern (Abb. 3). 0,7 % der Verstorbenen hatten zusätzlich zur Sepsis eine Entlassdiagnose für eine laborbestätigte Influenza.

Todesfälle traten nur zu einem sehr geringen Anteil aufgrund von Neugeborenensepsis oder schwangerschaftsassoziierter Sepsis auf (0,2 % bzw. 0,005 % der Sepsistodesfälle). Verstorbene mit septischem Schock waren jünger (medianes Alter 74 Jahre (IQR 17)), seltener weiblich (40,8 %) und hatten häufiger eine intensivmedizinische Komplexbehandlung erhalten (69,4 %, Tab. 1).

**Table 1:** 

	Sepsis (explizite Definition, inkl. septischen Schocks)	Septischer Schock (explizite Definition)	Sepsis (implizite Definition)
*Todesfälle im Jahr 2016*	58.689	25.111	196.440
*Alter, Mittelwert (SA), Median (IQR)*	72,7 (13,5), 76 (16)	71,4 (13,4), 74 (17)	76,1 (12,9), 78, (15)
*Weibliches Geschlecht (%)*	41,2	40,8	44,5
*Charlson-Komorbiditätsindex, Mittelwert (SA), Median (IQR)*	2,4 (1,5), 2 (2)	2,3 (1,5), 2 (2)	2,5 (1,5), 2 (2)
*Komorbiditäten nach Charlson-Komorbiditätsindex (%)*			
Diabetes	32,2	31,8	31,3
Chronische Lungenerkrankung	16,9	17,5	21,1
Nierenerkrankung	33,5	31,0	36,3
Herzinsuffizienz oder Myokardinfarkt	45,4	43,9	48,4
Krebs	22,8	22,5	24,0
Demenz oder zerebrovaskuläre Erkrankung	22,1	17,5	27,7
Lebererkrankung	10,9	12,2	8,2
HIV/Aids	0,2	0,2	0,1
*Infektionsfokus (%)*			
Respiratorische Infektion	52,5	53,2	59,3
Influenza (laborbestätigt)	0,7	0,9	0,4
Abdominelle Infektion	21,4	25,6	15,9
Wund‑/Weichteilinfektion	5,9	6,7	4,5
Urogenitalinfektion	24,3	21,1	26,0
ZNS-Infektion	1,3	1,2	0,8
Kardiovaskuläre Infektion	4,7	4,7	2,9
Fremdkörperassoziierte Infektion	8,1	8,4	4,7
Schwangerschaftsassoziierte Infektion	0,0	(^a^)	0,0
*Neonatale Sepsis (%)*	0,2	(^a^)	0,1
*Anteil septischer Schock (%)*	42,8	100,0	12,8
*Chirurgischer Eingriff im terminalen Aufenthalt (%)*	43,2	50,5	30,1
*Intensivmedizinische Komplexbehandlung im terminalen Aufenthalt (%)*	60,0	69,4	40,9
*Mechanische Beatmung im terminalen Aufenthalt (%)*	63,8	78,0	42,7
*Dauer mech. Beatmung in Stunden, Mittelwert (SA), Median (IQR)*	251,6 (381,7), 119 (288)	235,8 (369,2), 103 (270)	205,2 (330,9), 90 (232)
*Krankenhausliegedauer in Tagen, Mittelwert (SA), Median (IQR)*	17,0 (22,0), 10 (18)	16,6 (22,3), 10 (19)	14,4 (17,4), 9 (15)

*SA* Standardabweichung, *IQR Interquartile Range, ZNS* zentrales Nervensystem

^a^ Fallzahl < 3, daher keine Auswertung über die DRG-Statistik möglich

### Sepsismortalität und Anteil sepsisassoziierter Todesfälle unter allen Todesfällen

Die Mortalität der krankenhausbehandelten Sepsis betrug 73/100.000 Einwohner. Sie variierte 1,8-fach zwischen den Bundesländern (Minimum = 55, Maximum = 99, Median = 76/100.000 Einwohner, Tabelle S1, Onlinematerial) und 7,9-fach zwischen den Kreisen (Minimum = 24, Maximum = 189, Median = 69/100.000 Einwohner). Eine ähnliche Spannweite zeigte sich hinsichtlich der altersstandardisierten Sepsismortalität, die 2,0-fach zwischen 55 und 110/100.000 Einwohner zwischen den Bundesländern und 7,1-fach zwischen 24 und 171/100.000 Einwohner zwischen den Kreisen variierte.

**Figure 4:**
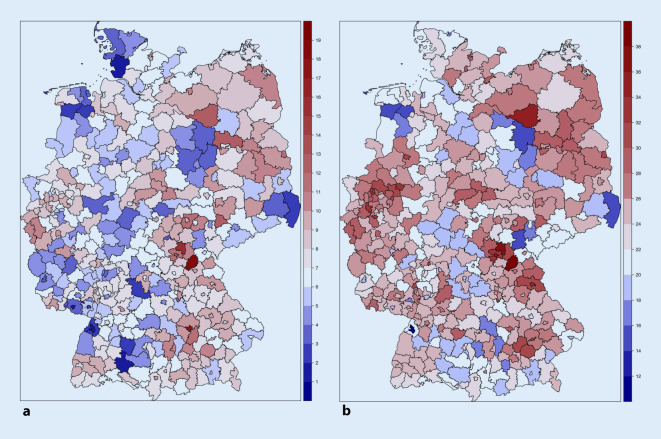

6,4 % der deutschlandweiten Todesfälle waren mit Sepsis assoziierte Krankenhaustodesfälle. 2,8 % der deutschlandweiten Todesfälle entfielen auf Krankenhausverstorbene mit septischem Schock. Der Anteil von sepsisassoziierten Krankenhaustodesfällen unter allen Todesfällen war am höchsten in der Altersgruppe der 40- bis 64-Jährigen (9,6 %) und höher bei Männern als bei Frauen (7,7 % vs. 5,2 %). Dies traf auch auf den septischen Schock zu. Der Anteil von sepsisassoziierten Krankenhaustodesfällen an allen Todesfällen variierte 7,2-fach zwischen den Kreisen (Minimum = 2,1 %, Maximum = 15,1 %, Median = 6,2 %, Abb. 4a).

### Implizit codierte Sepsis im Vergleich

1.236.502 krankenhausbehandelte Patienten erfüllten im Jahr 2016 die Kriterien für eine implizit definierte Sepsis (6,5 %). Die Krankenhaussterblichkeit dieser Patienten lag bei 15,9 % (*n* = 196.440), dies entspricht 47,2 % aller Krankenhaustodesfälle. Die demografischen und klinischen Charakteristika der mit impliziter Sepsis im Krankenhaus verstorbenen Patienten sind in Tab. 1 dargestellt.

Die Mortalität der implizit codierten Sepsis zeigte eine 1,7-fache Variation zwischen den Bundesländern (Minimum = 197, Maximum = 337, Median = 257/100.000 Einwohner) und eine 3,5-fache Variation zwischen den Kreisen (Minimum = 130, Maximum = 449, Median = 238; altersstandardisierte Mortalität: Minimum = 134, Maximum = 357, Median = 232/100.000 Einwohner).

Mit implizit codierter, krankenhausbehandelter Sepsis assoziiert waren 21,6 % aller deutschlandweiten Todesfälle in 2016. In der Altersgruppe der 65- bis 80-Jährigen lag der Anteil bei 26,8 %, während er in allen anderen Altersgruppen zwischen 18,1 % und 23,5 % rangierte. Der Anteil der Sepsistodesfälle mit implizit codierter Sepsis an den Gesamttodesfällen schwankte 2,6-fach zwischen den Kreisen (Minimum = 12,1 %, Maximum = 31,6 %, Median = 21,0 %, Abb. 4b).

## Diskussion

Die vorliegende Studie zeigt für das Jahr 2016, dass in Deutschland 14,1 % der Krankenhaustodesfälle und 6,4 % aller Todesfälle mit krankenhausbehandelter Sepsis nach expliziter Definition assoziiert waren. Der höchste Anteil von sepsisassoziierten Todesfällen fand sich in der Altersgruppe der 40- bis 64-Jährigen; hier waren fast 10 % aller deutschlandweiten Todesfälle mit krankenhausbehandelter Sepsis assoziiert. Ein Drittel der Sepsisverstorbenen hatte im terminalen Krankenhausaufenthalt keine intensivmedizinische Komplexbehandlung erhalten. Die zugrunde liegenden Infektionsfokusse unterschieden sich deutlich zwischen männlichen und weiblichen Verstorbenen; neonatale und maternale Sepsis lagen nur bei einem sehr geringen Teil der Verstorbenen vor. Identifiziert man Sepsis über implizite Codieransätze, das heißt das Vorhandensein eines ICD-10-GM-Codes für Infektion und Organversagen, erhöht sich der Anteil von mit krankenhausbehandelter Sepsis Verstorbenen an allen Todesfällen auf 21,6 %.

Der Anteil von mit explizit codierter Sepsis assoziierten Todesfällen an den deutschlandweiten Todesfällen liegt im Bereich der Ergebnisse von Studien mit Nutzung von expliziten Sepsisidentifikationsverfahren in multikausalen Todesursachenstatistiken. In Italien fand sich ein expliziter Sepsiscode in 6,3 % aller Totenscheine [16]. In den USA wurden explizite Sepsiscodes bei 6,0 % aller Todesfälle im Totenschein angegeben [5]. Dort ereigneten sich 87 % dieser Todesfälle im Krankenhaus. Der Anteil von mit implizit codierter Sepsis assoziierten Todesfällen liegt hingegen im Bereich der Schätzungen der Global-Burden-of-Disease Studie von etwa 20 % [3], wobei ebenfalls ein implizites Identifikationsverfahren genutzt wird. Während explizite Identifikationsverfahren in Krankenhausabrechnungsdaten die Häufigkeit von Sepsis unterschätzen (etwa 2,2-fach nach Schätzungen einer monozentrischen Validierungsstudie [12]), kann es bei der impliziten Strategie zu einer Überschätzung kommen, weil Infektion und Organdysfunktion nicht zwingend kausal verbunden sind [17]. Es ist aber wahrscheinlich, dass die Häufigkeit von sepsisassoziierten Todesfällen höher liegt, als in dieser Studie basierend auf den expliziten Sepsiscodes geschätzt.

Ob Sepsis die zugrunde liegende Todesursache der Patienten war, die während des Krankenhausaufenthalts mit Sepsis verstarben, lässt sich in unseren Daten nicht abschätzen. Das heißt sepsisassoziierte Todesfälle können entweder an oder mit Sepsis verstorben sein. Eine aktuelle US-amerikanische Studie fand, dass Sepsis die direkte Todesursache bei etwa zwei Drittel der Todesfälle war, bei denen Sepsis im terminalen Krankenhaus- oder Hospizaufenthalt vorlag [18]. Etwa ein Drittel dieser Patienten verstarb nach der Akutphase der Sepsis; allerdings hatte Sepsis nach Sicht der Autoren trotzdem bei über 40 % dieser Patienten zum Versterben beigetragen [18]. Damit weisen unsere Daten zumindest darauf hin, dass Sepsis eine der häufigsten Todesursachen in Deutschland ist. Im Vergleich waren 6,6 % aller Todesfälle in Deutschland auf einen Schlaganfall (2013) und 5,2 % bzw. 7,1 % aller Todesfälle bei Frauen bzw. Männern auf einen Herzinfarkt zurückzuführen [19]. Die Krankenhaussterblichkeit dieser Erkrankungen lag im Vergleich bei ca. 9,2–10,5 % bei Schlaganfall (in Krankenhäusern mit bzw. ohne Spezialeinheiten für Schlaganfallpatienten; [19]) und 11,3 % bei Herzinfarkt [20].

Zur Akutsterblichkeit der Sepsis kommt außerdem eine relevante Langzeitsterblichkeit der Patienten, für die eine durch epigenetische Veränderungen hervorgerufene anhaltende Immunsuppression und eine verstärkte Arteriosklerose nach Sepsis, die zum häufigen Auftreten kardiovaskulärer Ereignisse führt, verantwortlich gemacht werden [21]. Diese bleibt in unseren Analysen unberücksichtigt, trägt aber wesentlich zur Last an sepsisassoziierten Todesfällen bei. In einer systematischen Übersichtsarbeit und Metaanalyse wurde die postakute 12-Monats-Sterblichkeit auf 15 % geschätzt [22]. In Deutschland lag sie nach Ergebnissen eines monozentrischen Sepsisregisters bei 19 % im ersten Jahr nach Sepsis [23].

Auffällig sind die regionalen Unterschiede in der Sepsismortalität und im Anteil sepsisassoziierter Todesfälle, die auch nach Altersstandardisierung vorhanden bleiben. In einzelnen Kreisen waren bis zu 15,1 % aller Todesfälle mit expliziter, krankenhausbehandelter Sepsis assoziiert. Diese können einerseits durch eine höhere Sepsisinzidenz, andererseits durch eine höhere Sepsissterblichkeit in diesen Kreisen hervorgerufen werden. Sowohl Sepsisinzidenz als auch Sepsissterblichkeit werden durch das Vorliegen chronischer Erkrankungen [24], durch die Häufigkeit invasiver oder immunsuppressiver Therapien sowie durch sozioökonomische Faktoren beeinflusst [25]. Die genannten Faktoren können in Deutschland zwischen den Kreisen variieren und zu den hier beobachteten Unterschieden beitragen. Eine kausale Analyse ist durch das Fehlen von Daten der nicht im Krankenhaus behandelten Personen und personenbezogenen sozioökonomischen Informationen in der DRG-Statistik nicht möglich. Es ist nicht auszuschließen, dass auch Unterschiede im Codierverhalten zu diesen Unterschieden beitragen, da eine höhere Aufmerksamkeit auf die Erkrankung („Sepsis-Awareness“), beispielsweise durch Kampagnen und Schulungen, auch zu einer häufigeren Codierung von Sepsisfällen führen kann (sogenanntes Will-Rogers-Phänomen; [26]). Darauf weist hin, dass die regionalen Unterschiede bei impliziter Identifikation der Sepsisfälle deutlich geringer ausgeprägt sind als bei expliziter Erfassung (zum Beispiel 3,5-fache vs. 7,9-fache Variation der Sepsismortalität zwischen den Kreisen bei impliziter vs. expliziter Erfassung). Auch der Anstieg der mit explizit codierter Sepsis assoziierten Todesfälle, der sich im 10-Jahres-Intervall zwischen 26.606 Todesfällen in 2007 [8] und 58.689 Todesfällen in 2016 zeigt, kann mit diesen Faktoren zusammenhängen; zur wirklichen Bewertung dieses Phänomens bedarf es allerdings weiterer Forschung.

Die hier vorgestellte Studie bestätigt Beobachtungen vorheriger Studien, dass der Anteil sepsisassoziierter Todesfälle bei Männern und in mittleren Altersgruppen höher ist als bei Frauen bzw. bei hochaltrigen Patienten [5]. Beachtlich ist auch der hohe Anteil von Patienten mit explizit codierter Sepsis, die im terminalen Krankenhausaufenthalt keine intensivmedizinische Komplexbehandlung erhielten. Diese Patientengruppe ist bisher nur unzureichend erforscht; US-Studien legen nahe, dass diese Patienten älter sind, eine geringere Zahl von Organdysfunktionen haben und häufiger unter Infekten wie Harnwegsinfekten leiden [27]. Inwieweit Therapielimitierungen dazu beitragen, dass diese Patienten nicht auf Intensivstation behandelt werden, ist bisher unklar. Bei Patienten, die kurz nach Aufnahme auf die Intensivstation verstarben, kann aber auch die kurze Liegedauer dazu beitragen, dass der OPS-Code für intensivmedizinische Komplexbehandlung nicht codiert werden konnte, da die Mindestanforderung für die Codierung ein Intensivstationsaufenthalt von 24 h ist. Darüber hinaus müssen bestimmte Strukturvoraussetzungen erfüllt sein, um die OPS-Codes für eine intensivmedizinische Komplexbehandlung zu codieren, die möglicherweise nicht bei allen Krankenhäusern gegeben sind. Zu einem besseren Verständnis von Sepsistodesfällen ist es notwendig, auch die Gruppe nicht intensivmedizinisch behandelter Sepsispatienten verstärkt in Studien zu betrachten.

Unsere Studie hat Limitationen, die bei der Interpretation der Daten berücksichtigt werden müssen: Die Studie erfasst erstens ausschließlich krankenhausbehandelte Sepsisfälle und lässt daher keinen Rückschluss auf die Häufigkeit und den Anteil aller Sepsistodesfälle an den Gesamttodesfällen zu. Zweitens werden in unserer Studie nur Todesfälle als sepsisassoziiert erfasst, bei denen ein ICD-10-GM-Code für Sepsis (explizite Identifikationsstrategie) oder gleichzeitig ICD-10-GM-Codes für Infektion und Organversagen (implizite Identifikationsstrategie) bei Krankenhausentlassung vergeben wurden. Die niedrige Sensitivität der expliziten Sepsisdiagnosecodes kann zu einer Unterschätzung der Todesfälle führen [12]. Darüber hinaus kann drittens, wie bereits diskutiert, keine Aussage zur kausalen Todesursache gemacht werden. Viertens wurde im Erhebungsjahr dieser Studie (2016) die neue Sepsisdefinition (Sepsis-3) publiziert [1]. Auch wenn die entsprechende Änderung der S3-Leitlinie Sepsis und die Anpassung der klinischen Sepsis-ICD-Codes (R65.0 und R65.1) erst 2018 und 2020 erfolgten, kann nicht sicher ausgeschlossen werden, dass die neue Definition einen Einfluss auf die Diagnosekriterien der Sepsis in diesem Jahr (2016) hatte, was die Vergleichbarkeit zu den Vorjahren gegebenenfalls einschränkt.

## Fazit

Sepsisassoziierte Todesfälle sind häufig und stehen mit einem relevanten Anteil der Todesfälle in Deutschland in Verbindung, wenn auch unklar bleibt, wie häufig Sepsis die unmittelbare Todes*ursache* ist. Patienten, die an oder mit Sepsis versterben, sind häufig vorerkrankt, was die Notwendigkeit von Präventionsmaßnahmen für Sepsis in dieser Population unterstreicht, beispielsweise durch Impfungen. Es finden sich deutliche regionale Unterschiede im Anteil sepsisassoziierter Todesfälle an den Gesamttodesfällen auf Kreisebene, die bisher unzureichend verstanden sind und den Bedarf weiterer regionalisierter epidemiologischer Sepsisforschung, insbesondere unter Nutzung weiterer Datenquellen wie Krankenakten oder Register, nahelegen. Um sepsisassoziierte Todesfälle auch über die Todesursachenstatistiken abbilden und international vergleichen zu können, ist die Erweiterung der monokausalen hin zu einer multikausalen Statistik in Deutschland dringend notwendig.

## Supplementary Information




