# Supplementary material for: Sepsis-associated deaths in Germany: characteristics and regional variation
Source: Bundesgesundheitsblatt Gesundheitsforschung Gesundheitsschutz. 2021 Nov 8;65(3):388–95. [Article in German] doi: 10.1007/s00103-021-03427-5 (PMC8575348; doi:10.1007/s00103-021-03427-5)
Supplement: Supplementary file 1 [file 103_2021_3427_MOESM1_ESM.pdf]

Bundesgesundheitsblatt 4/2022

Onlinematerial zum Beitrag:

Sepsisassoziierte Todesfälle in Deutschland: Charakteristika und regionale Variation

Carolin Fleischmann-Struzek[1,2], Norman Rose[1,2], Konrad Reinhart[3]

[1] Institut für Infektionsmedizin und Krankenhaushygiene, Universitätsklinikum Jena, Jena, Deutschland
[2] Center for Sepsis Control and Care, Universitätsklinikum Jena, Jena, Deutschland
[3] Klinik für Anästhesiologie m. S. operative Intensivmedizin, Charité Universitätsmedizin Berlin, Berlin, Deutschland

Korrespondenzadresse:
Dr. med. Carolin Fleischmann-Struzek
Institut für Infektionsmedizin und Krankenhaushygiene
Universitätsklinikum Jena
Am Klinikum 1
07740 Jena, Deutschland
E-Mail: carolin.fleischmann@med.uni-jena.de

Inhalt:

- **Operationalisierungen basierend auf ICD-10-GM Codes und OPS-Codes**
- **Tabelle S1:** Fallzahlen, Inzidenz (roh, altersstandardisiert), Todesfälle und Mortalität (roh, altersstandardisiert) der explizit identifizierten Sepsis nach Bundesland in Deutschland in 2016

Operationalisierungen basierend auf ICD-10-GM Codes und OPS-Codes

<u>Infektfokus</u>

Respiratorische Infektionen: J01, J02, J03, J04, J06, J05, J09, J10, J11, J12, J13, J14, J15, J16, J17, J18, J20, J21, J22, J44.0, J44.1, J86, J85, A15, A16, U69.00, A36, A37, B38, B39

Abdominelle Infektionen: A00, A01, A02, A03, A04, A05, A06, A07, A08, A09, K35, K37, K36, K57.02, K57.03, K57.12, K57.13, K57.22, K57.23, K57.32, K57.33, K57.42, K57.43, K57.52, K57.53, K57.82, K57.83, K57.92, K57.93, K61, K65, K67, K63.0, K63.1, K75.0, K75.1, K81.0, K77.0, U69.40!

Wund-/Weichteilinfektionen: A46, B47, L03, L04, L08, L05, B00, B07, B08, B09, H05.0, H60.2, H70.0, J36, J39.0, J39.1, L02

Urogenitalinfektionen: N10, N15.1, N15.9, N34, N30, N39.0, N41, N45, N48.2, N49, N70, N71, N72, N73, N74, N75, N76, N77, N61, N98.0, A59, A55, A56

ZNS-Infektionen: A39, G00, G01, G02, G03, G04, G05*, G06, G07*, G08, A17+, A81, A83, A84, A85, A86, A87, A88, A89

Kardiovaskuläre Infektionen: I32, I33, I39, I40, I41, I80, I38, I98.1

Fremdkörper-assoziierte Infektionen: T82.6, T82.7, T83.5, T83.6, T84.5, T84.6, T84.7, T85.7

Schwangerschafts-assoziierte Infektionen: O75.3, O85, O03.0, O03.5, O04.0, O04.5, O05.0, O05.5, O06.0, O06.5, O07.0, O07.5, O08.0, O86, O23, O41.1, O88.3, O91, O98

Implizite Sepsis: o.g. Infektionen nach Fokus oder unspezifische Infektion: A18, A19, A20, A21, A22, A23, A24, A25, A26, A27, A28, A32, A38, A42, A43, A44, A48, A49, A54, A690, A691, A692, A698, A699, A97, B37, B40, B41, B42, B43, B44, B45, B46, B48, B49, B50, B51, B52, B53, B54, M00, M01, M86, T802, T814, T880, A90, A91, A50, A65, A74, A75, A77, A78, A79, A80, A92, A93, A94, A95, A96, A98, A99, B01, B02, B03, B04, B05, B06, B25, B26, B27, B33, B34, B55, B58, B60, B64, B67, B95, B96, B97, B98, B99

und Vorhandensein einer Organdysfunktion: I959, R578, R579, J96, J80, J984, R060, R068, F05, G931, G934, R40, N17, N19, E872, D65, D688, D689, D695, D696, K720, K727, K729, K762, K763, R651, R572

Neonatale Sepsis: P36

Influenza-Infektion (laborbestätigt): J09, J10

Septischer Schock: R57.2

Chirurgischer Eingriff: Entsprechend Variable DRG Statistik

Intensivmedizinische Komplexbehandlung: 8-980, 8-98d, 8-98f

Mechanische Beatmung: Entsprechend Variable DRG Statistik

Liegedauer: Entsprechend Variable DRG Statistik

Tabelle S1: Fallzahlen, Inzidenz (roh, altersstandardisiert), Todesfälle und Mortalität (roh, altersstandardisiert) der explizit identifizierten Sepsis nach Bundesland in Deutschland in 2016

*pro 100,000 Einwohner

Bundesland	Fälle	Todesfälle	Inzidenz*	AS Inzidenz*	Letalität	Mortalität*	AS Mortalität*
Baden-Württemberg	15558	6289	142	149	40,4	57	61
Bayern	26983	9309	209	218	34,5	72	76
Berlin	9266	3541	259	285	38,2	99	110
Brandenburg	4498	1935	180	161	43,0	78	69
Bremen	1385	631	204	208	45,6	93	95
Hamburg	3830	1630	212	238	42,6	90	103
Hessen	8334	3471	134	139	41,6	56	58
Mecklenburg-Vorpommern	4556	1503	283	256	33,0	93	83
Niedersachsen	12470	5209	157	155	41,8	66	65
Nordrhein-Westfalen	30479	13359	170	173	43,8	75	76
Rheinland-Pfalz	5537	2238	136	135	40,4	55	55
Saarland	2176	971	218	202	44,6	97	89
Sachsen	8628	3359	211	185	38,9	82	71
Sachsen-Anhalt	3794	1517	170	145	40,0	68	57
Schleswig-Holstein	4096	1680	142	137	41,0	58	56
Thüringen	5395	2047	250	220	37,9	95	83

Abkürzungen: AS = altersstandardisierte
